# Acquisition and consolidation processes following motor imagery practice

**DOI:** 10.1038/s41598-021-81994-y

**Published:** 2021-01-27

**Authors:** Célia Ruffino, Charlène Truong, William Dupont, Fatma Bouguila, Carine Michel, Florent Lebon, Charalambos Papaxanthis

**Affiliations:** Cognition, Action et Plasticité Sensorimotrice (CAPS), INSERM UMR1093, UFR STAPS, Université de Bougogne Franche-Comté, 21000 Dijon, France

**Keywords:** Neuroscience, Psychology

## Abstract

It well-known that mental training improves skill performance. Here, we evaluated skill acquisition and consolidation after physical or motor imagery practice, by means of an arm pointing task requiring speed-accuracy trade-off. In the main experiment, we showed a significant enhancement of skill after both practices (72 training trials), with a better acquisition after physical practice. Interestingly, we found a positive impact of the passage of time (+ 6 h post training) on skill consolidation for the motor imagery training only, without any effect of sleep (+ 24 h post training) for none of the interventions. In a control experiment, we matched the gain in skill learning after physical training (new group) with that obtained after motor imagery training (main experiment) to evaluate skill consolidation after the same amount of learning. Skill performance in this control group deteriorated with the passage of time and sleep. In another control experiment, we increased the number of imagined trials (n = 100, new group) to compare the acquisition and consolidation processes of this group with that observed in the motor imagery group of the main experiment. We did not find significant differences between the two groups. These findings suggest that physical and motor imagery practice drive skill learning through different acquisition and consolidation processes.

## Introduction

Motor learning is crucial for the accomplishment of numerous daily activities in a challenging environment. Through repetition, movements are executed faster, more accurately, and more effortlessly^1^. With extensive practice a new initially fragile motor memory is transformed into a robust and stable motor memory^2,3^. In the initial stage, significant improvement in skilled motor learning is observed during a single or a few training sessions (fast learning process). Its consolidation, however, is achieved gradually (slow learning process) through multiple training sessions. Interestingly, consolidation of skill motor behavior can be observed between practice sessions, that is without additional practice, commonly referred as off-line learning^2,4–8^. Several investigations have highlighted the fundamental role of sleep and the passage of time on off-line motor learning^4,9–11^. It appears that distinct mechanisms are engaged to support motor memory consolidation over wakefulness and sleep. Specifically, movement execution (i.e., speed and accuracy) is processed over wakefulness but not over sleep, whereas the spatial goal of a motor skill is processed over sleep but not wakefulness^10–12^.

Although physical practice is undeniably fundamental for the acquisition and consolidation of new skills, complementary methods have also been proposed^13,14^. Among them, motor imagery, a form of mental practice, is widespread^15–18^. Motor imagery is defined as the mental simulation of an action without any corresponding motor output. Several investigations have provided robust evidences that executed and imagined movements trigger similar motor representations. This is attested by several similitudes in their temporal organization^19–23^ and neural foundation, such as the activation of the parietal and prefrontal cortices, the supplementary motor area, the premotor and primary motor cortices, the basal ganglia, and the cerebellum^24^. In addition, it is now well-accepted that motor imagery training improves muscle strength^25–29^, flexibility^30^, speed and accuracy^16,31–34^ and provides a high promising method for motor rehabilitation^35–38^. The positive impact of motor imagery training on motor learning is associated with neural adaptations at several levels within the central nervous system^18,39^. Indeed, during the mental simulation of a movement, the generation of a subliminal motor command that triggers both cortical and spinal circuits is unmasked^40,41^. This activation induces plastic modulations, leading to gains in motor performance^28,39,42^.

Surprisingly, despite accumulated evidence about the consolidation processes during the daytime or after a night of sleep following physical practice, only few experimental data exist regarding skill consolidation after motor imagery training^43–45^. For instance, Debarnot and colleagues^40,41^ have eleganlty showed that sleep contributes to the consolidation of motor sequence learning acquired through motor imagery practice. Whether this finding is extended to different types of motor tasks, such as skilled motor learning, remained to be verified. In addition, the effects of the passage of time in motor skill consolidation has not been yet investigated.

Here, we used an arm pointing task emphasizing skill learning, namely a training-related change in the speed-accuracy tradeoff function. We assessed the immediate effects of physical or motor imagery training on skill acquisition (Post0h), as well as the effects of the passage of time (Post6h) and the effects of sleep (Post24h) on skill consolidation. During physical training, the advantage is that forward model predictions are compared with sensory feedback from the periphery, providing thus a better estimate of the state of the arm via a “self-supervised process” that minimizes prediction errors, namely the errors between the output from the forward model and the sensory outcome of the motor command^46,47^. Thus, after physical training, compared to mental training, we hypothesized a greater acquisition in skill learning. In addition, according to the literature showing that movement execution (i.e., speed and accuracy) is processed over wakefulness but not over sleep, whereas the spatial goal of a motor skill is processed over sleep but not wakefulness^10–12^, we also expected an effect of the passage of time but not sleep after both interventions.

## Main experiment

### Methods

#### Participants

Forty-two right-handed adults, without neurological, mental or physical disorders and with normal or corrected-to-normal vision, were randomly assigned into three groups: (i) the physical training group (PT, n = 14, 6 females, mean age: 22 ± 3 years old), the motor imagery training group (MIT, n = 14, 7 females, mean age: 24 ± 3 years old), and the control group (CT, n = 14, 5 females, mean age: 23 ± 2 years old).

Motor imagery ability of the MIT group was assessed by the French version of the Movement Imagery Questionnaire “MIQr”^48^. The MIQr is an 8-item self-report questionnaire, in which the participants rate the vividness of their mental images using two 7-point scales, one associated to visual and the other to kinesthetic imagery. The score ‘7′ indicates really easy to feel/visualize, whereas the score ‘1′ corresponds to really difficult to feel/visualize. The average score obtained here (46.12 ± 5.02) indicate good imagery ability (maximum score: 56; minimum score: 8).

All participants were synchronized with a normal diurnal activity (7 a.m. to 12 a.m.) alternating with a night sleep. None of the participants had a daily nap or a physical activity during this period. The chronotype of the participants, evaluated by the Morningness-Eveningness Questionnaire^49^, was moderate morning type (n = 4), moderate evening (n = 5) or neither type (n = 33). There were no significant differences between groups regarding their chronotype (PT: 47.29 ± 10.24; MIT: 51.43. ± 8.32; CT: 48.93 ± 12.75; one factor ANOVA, F_2,39_ = 0.50, *p* = 0.60).

The quality of participants’ sleep was evaluated by the Pittsburgh sleep quality index^50^. The general score in this questionnaire ranges from 0 (no particular difficulties to sleep) to 21 (major difficulties to sleep). All participants showed good sleep quality; individuals scores ranged from 1 to 9. Group averages were almost similar (PT: 4.50 ± 1.59; MIT: 4.79 ± 2.40; CT: 4.79 ± 1.93) and statistically not different (one factor ANOVA: F_2,39_ = 0.09, *p* = 0.92).

The experimental design was approved by the regional ethic committee (Comité de Protection des Personnes—Région EST) and was conformed to the standards set by the Declaration of Helsinki. All participants provided written informed consent after being informed on the experimental procedures.

#### Experimental device

**Figure 1:**
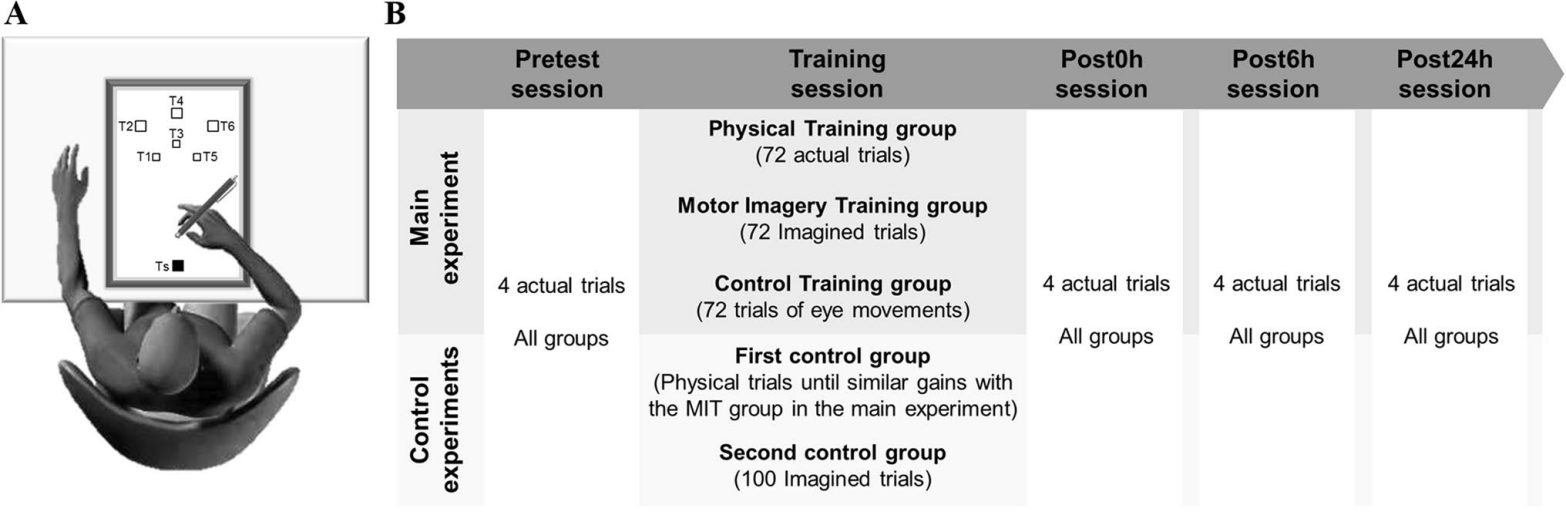
(**A**) Participants’ position and location of the targets on the graphic tablet. One trial comprised 12 successive point-to-point arm movements between the targets in the following order: Ts-T1-Ts-T2-Ts-T3-Ts-T4-Ts-T5-Ts-T6-Ts. The participants, using a pencil held in their right hand, were requested to point between the targets as accurately and as fast as possible. (**B**) Experimental procedure. The whole protocol comprised 5 sessions: Pretest, Training, Post0h (immediately after training), Post6h (6 h after training), and Post24h (1 day after the Pretest session). In the training session of the main experiment, the Physical Training group executed 72 actual trials, the Motor Imagery Training group accomplished 72 mental trials, and the Control Training group performed 72 trials by moving only the eyes between the targets. In the first control experiment, the participants executed physical trials to obtain the same gain than the Motor Imagery Training group of the main experiment. In the second control group, the participants accomplished 100 mental trials. In the other sessions, all participants executed 4 trials.

The participants were comfortably seated on a chair in front of a graphic tablet (Intuos4 XL, Wacom, Krefeld, Germany). Seven square targets were designed on it (see Fig. 1A). The target Ts (starting target, 1 × 1 cm) was aligned with the participants’ sternum at a distance of 10 cm. The other six targets had varying sizes and were placed at different distances from the Ts to vary the difficulty of the task within a trial (T1: 0.5 × 0.5 cm, 31 cm; T2: 1 × 1 cm, 38.5 cm, T3: 0.5 × 0.5 cm, 34 cm; T4: 1 × 1 cm, 42 cm; T5: 0.5 × 0.5, 31 cm; T6: 1 × 1 cm, 38.5 cm). One trial included 12 successive visually guided point-to-point arm movements between the targets in the following order: Ts-T1-Ts-T2-Ts-T3-Ts-T4-Ts-T5-Ts-T6-Ts. The participants, holding a pencil with their right hand, were requested to point between the targets as accurately and as fast as possible. Thus, the success of the task required a compromise between speed and accuracy. We notified the participants that all targets had to be reached in the specified order. No further instructions concerning motor performance (i.e., movement velocity or spatial accuracy) was provided to the participants during the whole protocol.

### Experimental procedure

The experimental protocol included five sessions (see Fig. 1B). The *pretest* session was scheduled in the morning between 8:30 a.m. and 9:00 a.m. All the participants accomplished 4 trials as accurately and as fast as possible. To warm up and familiarize themselves with the task, participants performed two trials before the *pretest* trials (one at a natural speed and another one as fast as possible). The *training* session started 3 min after the *pretest* session. The participants of the PT group carried out 72 actual trials as accurately and as fast as possible. Those of the MIT group imagined 72 trials as accurately and as fast as possible, combining kinesthetic and visual (first-person perspective) modalities^51^. To ensure that all participants of the MIT group would correctly complete the training phase, we provided the following instructions: ‘*try to imagine yourself performing the motor task, by seeing and feeling your arm moving as if you were actually moving it’*. In addition, we asked the participants to report the quality of each imagined trial on a 7-point-scale (1 = very poor; 7 = excellent). The grand average score (5.18 ± 0.98) indicates a successful motor imagery training session. The participants of the CT group were trained to only move their eyes between the targets. As the motor task required eye-hand coordination, an enhancement in motor performance after MIT could be attributed either to eye movement training or to a cognitive-attentional effect. By including the CT group, we controlled for such a bias in the improvement of motor skill^16^. The experimenter counted the number of eye movements between the 12 targets during each trial. Very few trials (< 1%, n = 1008, 72 trials × 14 participants) did not comprise 12 eye movements. Note that as our experimental protocol was standardized, training durations were almost similar between groups. Considering individual differences, the training phase lasted between 30 and 35 min.

The first *posttest* session (Post0h) started 3 min after the training session. The second *posttest* session (Post6h) was carried out 6 h later the same day, and the last *posttest* session (Post24h) was completed one day after the pretest session. The three posttest sessions were identical to the pretest session; that is, all participants carried out 4 actual trials as accurately and as fast as possible after two warm up trials.

The different post-test sessions allowed us evaluating: the immediate effects of training (acquisition) on motor performance (delta Pre_Post0h), the effects of the passage of time on skill consolidation (delta Post0h_Post6h), and the effects of sleep on skill consolidation (delta Post6h_Post24h).

### Kinematics recording and analysis

The graphic tablet enabled the recording of movement kinematics at 100 Hz. The spatial resolution in the present experiment was less than 1 mm. Data processing was performed by using custom software written in Matlab (Mathworks, Natick, MA). Recorded position signals in the horizontal plane (X, Y) were lowpass filtered using a digital fifth-order Butterworth filter with zerophase distortion (Matlab ‘butter’ and ‘filtfilt’ functions) at a cut-off frequency of 10 Hz.

In our task, movement execution was characterized by two parameters: duration and accuracy. Ascertaining, however, that motor skill (i.e., the training-related change in the speed-accuracy trade-off function) has been improved is not possible when duration and accuracy change in opposite directions^52^. For that reason, we computed a composite ratio of duration and accuracy to describe motor skill. Therefore, for each trial, we recorded the total movement duration, the spatial accuracy (rate of error), and the motor skill.

Movement duration (in seconds) was calculated as the time interval between the beginning of the trial (when the tangential velocity of the first movement, i.e., TS-T1, increased above zero) and the end of the trial (when the tangential velocity of the last movement, i.e., T6-Ts, returned to zero).

Error rate was defined as the percentage of the missed targets (when the participants didn’t touch the target at all) during a trial

$$\frac{nb\; of\; errors}{{12}} \times 100$$

where *nb of errors* is the number of missed targets during a trial, and *12* is the number of targets.

Motor skill was calculated as follows:

$$\frac{{1 - \left( {nb\frac{errors}{{12}}} \right)}}{duration}$$

where *duration* is the total movement duration of a trial, *nb of errors* is the number of missed targets during a trial, and *12* is the number of targets. Note that when the ratio increases, skill improves.

The gains were calculated to present, for each parameter, a positive gain (i.e., performance improvement) as follows:

i) For error rate:
  Gain Pre_Post0h = Pretest – Post0h
  Gain Post0h_Post6h = Post0h – Post6h
  Gain Post6h_Post24h = Post6h – Post24h
ii) For duration:
  Gain Pre_Post0h $$= \frac{Pretest - Post0h}{{Pretest}} \times 100$$
  Gain Post0h_Post6h $$= \frac{Post0h - Post6h}{{Post0h}} \times 100$$
  Gain Post6h_Post24h $$= \frac{Post6h - Post24h}{{Post6h}} \times 100$$
iii) For motor skill:
  Gain Pre_Post0h $$= \frac{Post0h - Pretest}{{Pretest}} \times 100$$
  Gain Post0h_Post6h $$= \frac{Post6h - Post0h}{{Post0h}} \times 100$$
  Gain Post6h_Post24h $$= \frac{Post24h - Post6h}{{Post6h}} \times 100$$

### Electromyographic recording and analysis

To verify that participants of the MIT group did not activate their muscles during motor imagery training, electromyographic (EMG) activity of the biceps brachii (BB) and the triceps brachii (TB) of the right arm was recorded during each imagined trial and compared to EMG activity at rest (10 s recording before training)^53^. We used pairs of bipolar silver chloride circular (recording diameter of 10 mm) surface electrodes positioned lengthwise over the middle of the muscles belly with an interelectrode (center to center) distance of 20 mm. The reference electrode was placed on the medial elbow epicondyle. After shaving and dry-cleaning the skin with alcohol, the impedance was below 5 kΩ. EMG signals were amplified (gain 1000), filtered (with a bandwidth frequency ranging from 10 Hz to 1 kHz), and converted for digital recording and storage with PowerLab 26 T and LabChart 7 (AD Instruments). We analyzed the EMG patterns of the muscles by computing their activation level (RMS, root mean square) using the following formula:

$$RMS = \sqrt {\frac{1}{MD}\mathop \smallint \limits_{0}^{MD} \left( {EMG} \right)^{2} dt}$$

### Statistical analysis

We first verified the normality of the data (Shapiro–Wilk test), the equality of variance (Levene’s test), and sphericity (Mauchly’s test). For error rate, movement duration, and motor skill, we performed repeated measures ANOVA and Newman-Keuls *post-hoc* when necessary with *group* as between-subject factor (PT, MIT, CT) and *session* as within-subjects factor (Pretest, Post0h, Post6h, Post24h). Gains (Pre_Post0h, Post0h_Post6h, and Post6h_Post24) were compared with the reference value *zero* (0) and by two-tailed *t-tests* for independent samples between groups. EMG signals at rest and during motor imagery training were compared by means of Wilcoxon tests (normality was violated). Statistical analysis was performed using STATISTICA (8.0 version; Stat-Soft, Tulsa, OK). The significance level was fixed at 0.05 and power was superior than 0.9 for all statistical analyses.

## Results

Table 1 shows the average values of error rate, movement duration, and motor skill for the three groups (PT, MIT, CT) and the four sessions (Pretest, Post0h, Post6h, Post24h), separately.

**Table 1:** Average values (+ SD) of Error Rate (%), Duration (s) and Skill (a.u.) at Pretest, Posttest 0 h, Posttest 6 h and Posttest 24 h for all groups for the main experiment (PT: Physical Training Group, MIT: Motor Imagery Training group, CT: Control Training group).

Groups		Pretest	Posttest 0 h	Posttest 6 h	Posttest 24 h
Error Rate (%)					
PT	Mean	8,63	11,66	10,27	9,67
	*SD*	*0,08*	*0,07*	*0,08*	*0,09*
MIT	Mean	11,01	11,11	9,52	10,66
	*SD*	*0,05*	*0,07*	*0,06*	*0,06*
CT	Mean	8,04	9,52	9,67	9,08
	*SD*	*0,04*	*0,06*	*0,07*	*0,05*
Duration (s)					
PT	Mean	7.95	6.94	7.10	7.11
	*SD*	*1.26*	*1.38*	*1.15*	*1.24*
MIT	Mean	7.76	7.36	7.08	7.07
	*SD*	*1.57*	*1.26*	*1.06*	*1.18*
CT	Mean	8.01	7.92	7.94	7.87
	*SD*	*0.93*	*0.97*	*0.81*	*0.77*
Skill (a.u)					
PT	Mean	0.117	0.131	0.129	0.130
	*SD*	*0.015*	*0.021*	*0.016*	*0.18*
MIT	Mean	0.118	0.123	0.130	0.129
	*SD*	*0.019*	*0.017*	*0.015*	*0.018*
CT	Mean	0.116	0.115	0.115	0.116
	*SD*	*0.010*	*0.010*	*0.012*	*0.010*

### Error rate

**Figure 2:**
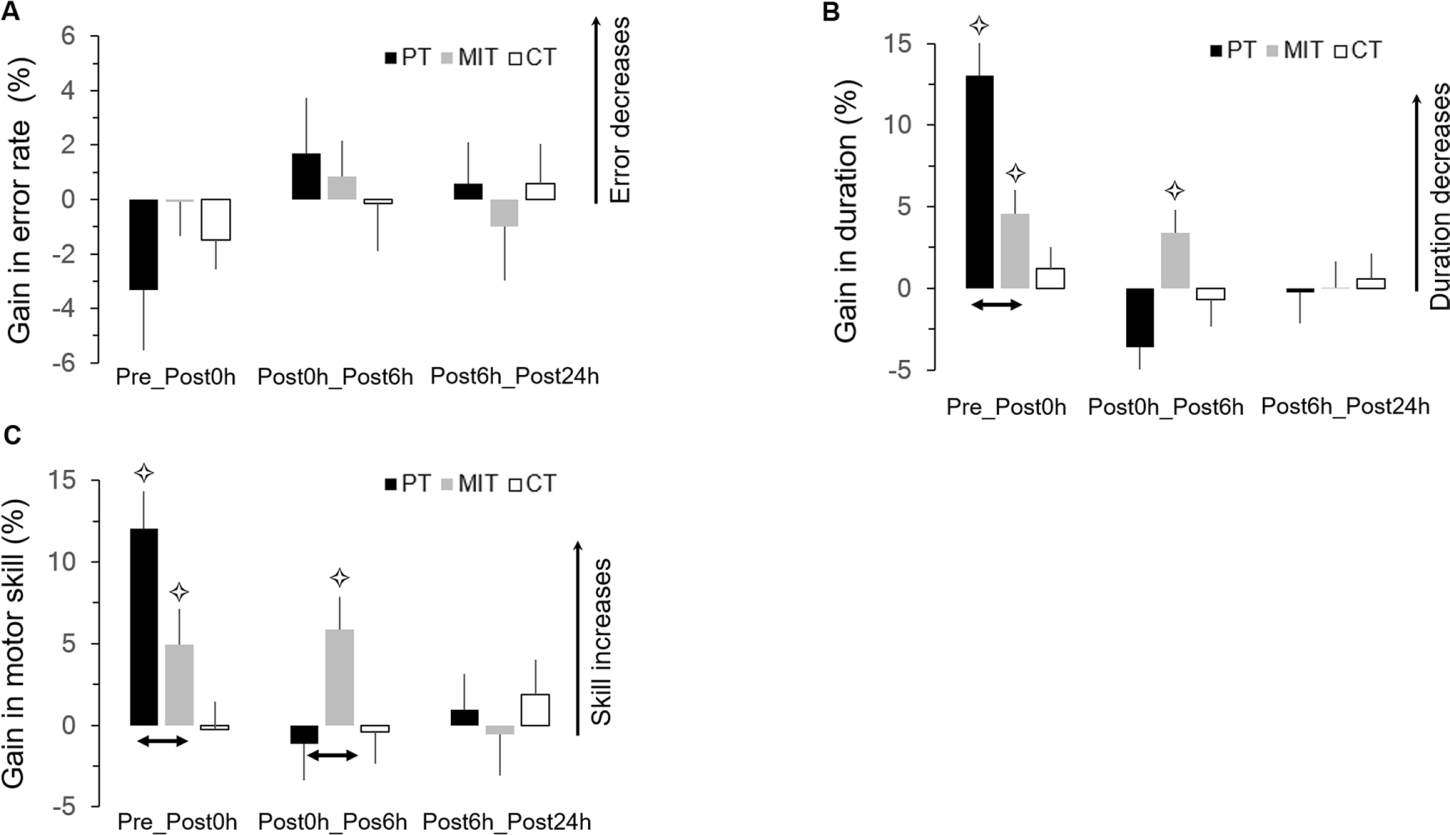
Average values (+ SE) of gains (%) in error rate (**A**), movement duration (**B**), and motor skill (**C**) between sessions for the three groups. Stars indicate significant differences from the value *zero* (0). Arrows indicate significant differences between groups. PT, Physical Training; MIT, Motor Imagery Training; C, control group.

All participants respected our requirements for accurate arm movements as the average error rate in all sessions were less than 12% (see Table 1). Neither training nor passage of time or sleep significantly enhanced or deteriorated spatial accuracy. rmANOVA did not reveal main (*group*: F_2,39_ = 0.256, *p* = 0.77; *session*: F_3,6_ = 0.803, *p* = 0.50) or interaction (F_6,117_ = 0.484, *p* = 0.82) effects. This result is further illustrated in the Fig. 2A, which shows the average gains (+ SE) in spatial accuracy. It appears that fluctuations in error rate were minimal between sessions (on average < 4%). The comparison (*t-tests*) of each gain with the reference value *zero* (0) did not reveal significant differences (in all, − 0.77 < t < 1.5, *p* > 0.13). Note that a second analysis, in which we calculated constant errors, i.e., the Euclidian distance of the end of each pointing movement to the center of the corresponding target, showed qualitatively similar results with those described above.

### Movement duration

On average, participants accomplished trials in less than 8.1 s (see Table 1). Movement duration significantly decreased between sessions for the PT and MIT groups, but not for the CT group. rmANOVA revealed an interaction effect (F_6,117_ = 3.985; *p* = 0.001; η^2^ = 0.17). *Post-hoc* analysis did not show significant differences among the three groups in the pretest session (in all, *p* > 0.7). Furthermore, movement duration for the CT group did not change across sessions (in all, *p* > 0.4), suggesting that eye movements did not improve arm movement speed. On the contrary, movement duration significantly decreased between the pretest session and each of the three posttest sessions (Post0h, Post6h, Post24h) for both PT and MIT groups (*p* < 0.02, for all comparisons).

Figure 2B illustrates the average gains (+ SE) in movement duration. Gains comparison (*t-tests*) with the reference value *zero* (0) showed significant differences for the Pre_Post0h gain of the PT group (t = 5.86; *p* < 0.001), as well as for the Pre_Post0h gain (t = 2.96; *p* = 0.01) and the Post0h_Post6h gain (t = 2.30; *p* = 0.03) of the MIT group. All the other comparisons did not reach the significant level (t < 1.45 and *p* > 0.17). This finding denotes that while movement speed was not further improved after the Post0h for the PT group, it was improved for the MIT group (Post6h). Thus, a positive effect of the passage of time could be inferred only for the MIT group. Note, that the Pre_Post0h gain was greater in the PT than in the MIT group (t = 3.14; *p* = 0.004), indicating the superiority of physical practice for movement speed improvement immediately after the training session. This immediate advantage, however, reversed at Post6h, because the Post0h_Post6h gain of the MIT group was almost significantly greater compared to that of the PT group (t = -− 1.97; *p* = 0.058). For both PT and MIT groups, gains in movement duration were consolidated at Post24h, without any off-line learning due to sleep (i.e., no further improvement in movement speed between Post6h and Post24h).

### Motor skill

The lower row of Table 1 shows the average values of motor skill. rmANOVA revealed a significant interaction effect (F_6,117_ = 3.738; *p* = 0.002; η^2^ = 0.16). Briefly, there were no significant effects between groups in the pretest session (in all, *p* > 0.8) nor between sessions for the CT group (in all, *p* > 0.7). Skill significantly improved between the pretest and each of the three posttest sessions (Post0h, Post6h, Post24h) for both PT and MIT groups (*p* < 0.01, for all comparisons). Figure 2C illustrates the average gains (+ SE) in motor skill. Gain comparisons (*t tests*) with the reference value *zero* (0) revealed significant differences for the Pre_Post0h gain of the PT group (t = 5.07; *p* < 0.001), as well as for the Pre_Post0h gain (t = 2.18; *p* = 0.04) and the Post0h_Post6h gain (t = 2.85; *p* = 0.02) of the MIT group. Pre_Post0h gain was greater in the PT than in the MIT group (t = 2.15; *p* = 0.04). On the contrary, Post0h_Post6h gain in the MIT group was significantly greater compared to PT group (t = -2.11; *p* = 0.04). For both PT and MIT groups, gains in movement speed were consolidated at Post24h, without any off-line learning due to sleep (i.e., no further improvement in movement speed between Post6h and Post24h).

**Figure 3:**
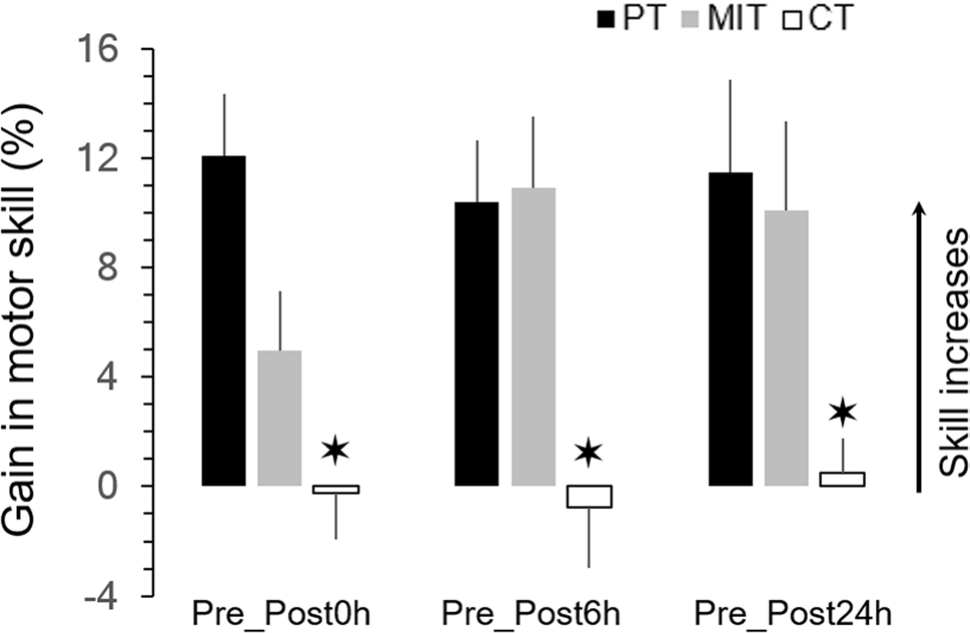
Average values (+ SE) of gains (%) in skill performance between the pretest and the posttest sessions. Star indicates significant differences between the control group and the two other groups (PT and MIT).

Figure 3 shows the average gains (+ SE) in skill with respect to pretest. It can be observed that the initial worse skill acquisition after motor imagery training compared to physical training (see Pre-Post0h; difference between PT and MIT groups = 7.11%) is compensated by the passage of time (see Pre_Post6h, difference between PT and MIT groups = − 0.52%). The skill of the CT group did not show any improvement. rmANOVA (two-way: 3 groups × 3 gains) revealed a significant effect of group (F_2,39_ = 8.05; *p* < 0.001; η^2^ = 0.31). *Post-hoc* comparisons showed that the skill of PT and MIT significantly differed from that of the CT group (for both, *p* < 0.001).

### Electromyographic analysis

Participants did not activate their muscles during motor imagery training. The grand average of EMG activity during imagined trials was 0.003 mV ± 0.002 and comparable to that recorded at rest (0.003 mV ± 0.001). Statistical comparison (Wilcoxon tests) of EMG activity between each imagined trial and the rest revealed no significant difference neither for the BB muscle (for all Z < 1; *p* > 0.50) nor for the TB muscle (for all, Z < 1; *p* > 0.50). Note that a more detailed analysis, using a moving window of 1 s for each imagined trial, did not show significant differences in EMG activity between imagined and rest trials (in all, *p* > 0.1).

## Control experiments

In the main experiment, we found that motor skill progressively enhanced between Post0h and Post6h for the MIT group, but not the PT group. This finding may suggest a different consolidation process between the two groups. Before considering such a premise, however, one must look for differences in the acquisition processes that could influence the subsequent consolidation process. Indeed, although both groups received the same amount of practice (i.e., 72 trials), motor performance of the PT group was significantly greater compared to that of the MIT. One can hypothesize that skill could improve after physical practice if the amount of learning was similar to that after motor imagery practice.

This was the aim of the first control experiment. We tested nine new participants (4 females, mean age: 24 ± 2 years old) in the same motor task as described above. After accomplishing 4 actual trials as accurately and as fast as possible (pretest session), the participants started the training session. Their skill performance was controlled online. As the average gain in skill performance reached by the MIT group was 4.95% (see Fig. 2C), the physical training ended when the skill gain reached 5%. The other sessions (Post6h and Post24h) were similar to the main experiment. Data analyses and statistical comparisons were also analogous with those of the main experiment.

**Figure 4:**
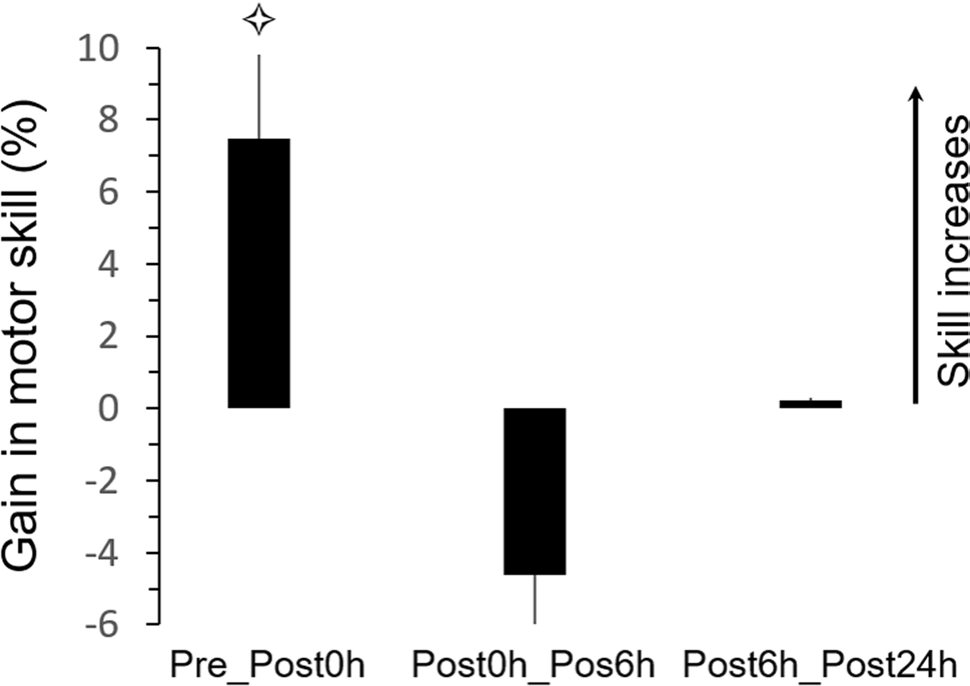
Control Experiment 1. Average values (+ SE) of gains (%) in motor skill between sessions. Stars indicate significant differences from the value *zero* (0).

The average values (+ SD) of skill performance for the different sessions were: 0.121 ± 0.011 (Pretest), 0.130 ± 0.011 (Post0h), 0.123 ± 0.012 (Post6h), and 0.123 ± 0.010 (Post24h). One-way Anova showed a significant effect of *session* (F_3,24_ = 3.42; *p* = 0.003; η^2^ = 0.30). *Post-hoc* analysis showed significant differences between the Pretest and Post0h sessions (*p* = 0.027) and between the Post0h and Post6h session (*p* = 0.037). Figure 4 illustrates the average gains (+ SE) in motor skill. This latter significantly improved by + 7.46% after training (Pre_Post0h gain compared to reference value *zero* (0); t = 8.97; *p* < 0.001); note that skill improvement for the PT and MIT group in the main experiment was 12.07% and 4.95%, respectively. Interestingly, skill performance decreased, although not significantly, at Post6h by -4.60% (Post0h_Post6h gain compared to reference value *zero* (0); t = -− 1.62; *p* = 0.14) and stabilized at Post24h (0.23%; Post6h_Post24h gain compared to reference value *zero* (0); t = 0.10; *p* = 0.91). These findings are opposite to those of the MIT group, for which an increase in motor skill was observed with the passage of time. Thus, for almost similar gains during motor acquisition (+ 7.46% for physical training in the control experiment and 4.95% for motor imagery training in the main experiment), the consolidation process differed between the two training methods.

In the second control experiment, our aim was twofold: to test whether we could obtain similar amount of skill acquisition with that observed after physical training in the main experiment and to observe eventual changes on the acquisition and consolidation processes. We included ten new participants (2 females, mean age: 25 ± 3 years old) in the same motor task as described above. Participants carried out 100 imagined trials during the training session. The other sessions (Prestest, Post6h and Post24h) were similar to the main experiment. Data analyses and statistical comparisons were also analogous with those of the main experiment.

**Figure 5:**
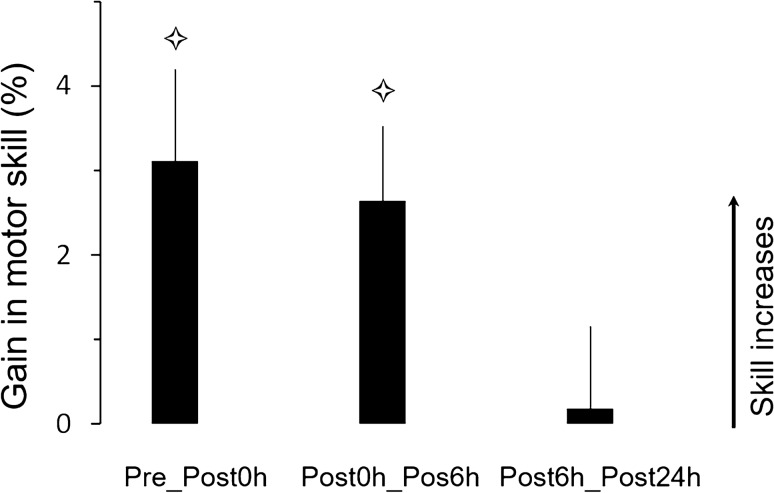
Control Experiment 2. Average values (+ SE) of gains (%) in motor skill between sessions. Stars indicate significant differences from the value *zero* (0).

Qualitatively, our results were similar with those of the main experiment. Precisely, we found that motor skill increased at Post0h and Post6h and stabilized at Post24h. The average values (+ SD) of skill performance for the different sessions were: 0.116 ± 0.346 (Pretest), 0.120 ± 0.338 (Post0h), 0.123 ± 0.342 (Post6h), and 0.123 ± 0.391 (Post24h). One-way Anova showed significant effect of *session* (F_3,27_ = 3.42; *p* = 0.0004; η^2^ = 0.48). *Post-hoc* analysis showed significant differences between the Pretest and all the other sessions (in all, *p* < 0.03) and between the Post0h and the other sessions (in all, *p* < 0.05). Figure 5 illustrates the average gains (+ SE) in motor skill. This latter significantly improved by + 3.11% after training (Pre_Post0h gain compared to reference value *zero* (0); t = 2.37; *p* = 0.04), by + 2.64% at Post6h (Post0h_Post6h gain compared to reference value *zero* (0); t = 2.31; *p* = 0.04) and stabilized at Post24h (+ 0.18%; Post6h_Post24h gain compared to reference value *zero* (0); t = 0.24; *p* = 0.81).

## Fatigue measurements

We evaluated the participants’ fatigue because our protocols included an important number of movements per group (n = 864; 72 trials × 12 arm movements for the PT, MIT and CT groups of the main experiment; n = 1200; 100 trials × 12 arm movements for the control group 2). The participants reported their mental fatigue after the training session, by means of a 200 mm visual analog scale (0 mm: ‘no fatigue’, 200 mm ‘maximal fatigue’). Group averages were almost similar (PT: 84 ± 51; MIT: 77 ± 49; CT: 76 ± 47; Control 2: 69 ± 47) and statistically not different (one factor ANOVA: F_3,48_ = 0.19, *p* = 0.89).

## Discussion

In the current study, we examined motor skill acquisition and consolidation after motor imagery or physical training in a speed/accuracy trade-off task. We observed that although skill significantly improved immediately after both practices, acquisition was higher after physical practice. Interestingly, we found a positive impact of the passage of time on skill consolidation (off-line improvement) only after motor imagery training. Sleep did not promote skill consolidation for any of the interventions. Overall, our findings suggest that physical and motor imagery practices drive motor skill learning through different acquisition and consolidation processes.

### Skill acquisition

In our study motor performance is limited by the trade-off between speed and accuracy. Therefore, skill development required either changes at both variables in the expected direction (i.e., more accurate and faster) or changes of at least in one variable in the expected direction (i.e., more accurate or faster) with the other variable remaining stable (same speed or same accuracy, respectively).

Like the participants of the PT group, those of the MIT group significantly enhanced their skill performance immediately after the training session. This finding corroborates and expands those of previous studies, which reported significant improvement in motor performance after motor imagery practice^16,31–34,54,55^. The lack of skill improvement for the CT group further supports the specific benefits of motor imagery training on motor behavior. Here, gains in skill performance for both training groups were obtained by mainly increasing movement speed. This finding qualitatively indicates a common acquisition process for the two practice methods. Note that similar results have also been observed on an arm pointing task. Precisely, Debarnot et al. (2009), using a computer mouse, instructed the participants to point from a central target toward peripheral targets. The results revealed that participants performed the sequence more rapidly, without improving the accuracy of their movements.

Not surprisingly, the PT group showed higher gains in skill than MIT group immediately after practice. This immediate superiority in motor acquisition of physical practice compared to mental practice is a constant finding in the literature^16,25,32–34,56^. Theoretically, the concept of internal forward models can explain this result^16,33,56^. Evidence support the hypothesis that motor prediction is generated by internal forward models, which are neural networks that mimic the causal flow of the physical process by predicting the future sensorimotor state (e.g., position, velocity) given the efferent copy of the motor command and the current state^57^. A recent study has elegantly showed that a forward model is triggered to predict the sensory consequence of the imagined movement^58^. During motor imagery practice, the initial state of the arm and a copy of the motor command (efferent copy) generated by the controller are both available to the forward model, which makes predictions for the future states of the arm. These internal predictions, which are the inputs of the forward model to the controller, could improve the output of the controller in the absence of movement-related sensory feedback, and therefore enhance motor performance in a subsequent motor task. However, although forward internal models are engaged during motor imagery, sensorimotor prediction is more variable, since it is not updated by sensory feedbacks, which may explain the difference between PT and MIT groups^59^. Recent findings have demonstrated that motor imagery-based practice drives motor learning of a kinematically complex multi-articular motor skill in the absence of sensory feedback^60^. During physical training, the advantage is that forward model predictions are compared with sensory feedback from the periphery, providing thus a better estimate of the state of the arm via a “self-supervised process” that minimizes prediction errors, namely the errors between the output from the forward model and the sensory outcome of the motor command^46,47^. Thus, under physical training, compare to mental training, one can hypothesize a better functional linkage between the forward model and the controller, leading to better skill learning. The fact that increasing the number of imagined trials (control experiment 2) within a single training session did not induce better acquisition further indicates that differences in acquisition between physical and motor imagery practice are not due to the quantity of training, but rather to functional process associating internal predictions with sensory feedback. However, it must be noted that multiple-sessions training with motor imagery could induce further improvements in skill performance to that obtained after physical practice.

When considering skill acquisition process, it is important to examine both temporal and spatial components of the task. In our study, we observed significant differences between PT and MIT groups for movement speed (i.e., temporal component) but not error rate (i.e., spatial component). More interestingly, while the PT group showed a small, although not significant, deterioration of error rate after training (approximatively -3%), the MIT group showed stabilization. This may reveal that skill learning does not equally involve spatial and temporal components in the two practices. Several research groups explored the difference between spatial (i.e., error rate) and temporal (movement duration) components^61–64^. They described that MI engenders learning that is more spatial than temporal in nature. Future studies, emphasizing more the spatial component of the motor task, could bring further information on the nature of skill performance improvement between physical and motor imagery practices.

### Skill consolidation

Previous studies have shown that physical practice lead to off-line gains in motor performance after a day time interval and/or a period of sleep^2,4,5,10,12,65,66^. For instance, on a visuomotor adaptation paradigm, several authors observed a positive effect of a retention interval on offline learning processes, with a strong resistance to interference^67,68^. Here, we showed that a single session of motor imagery training, like physical training, lead to a long-term motor memory formation. Indeed, motor skill was significantly improved one-day after the motor imagery training session (Post24h versus Pretest). This result corroborates and expands findings from previous studies, which showed permanent changes in motor behavior after mental training^16,28,34,43,69^.

Skill improvement after physical or mental practice is, however, processed off-line over different brain states. Precisely, the passage of time enhanced motor skill after motor imagery training only (off-line learning at post6h), while sleep did not induce any offline learning (Post24h) whatever the practice method. Previous studies have shown that motor consolidation is related to the nature of the task, and more specifically to the explicit or implicit components composing it. Indeed, the spatial goal of a movement (explicit component) is processed over sleep but not during wakefulness, whereas the skilled movements are processed over wakefulness but not over sleep^10,12^. Our arm pointing task required skilled movements without any explicit learning; the goal of the task was to reach all the targets in the same predetermined order. Therefore, based on the above described previous findings, we expected an effect of the passage of time on skill. This hypothesis was verified for motor imagery training, but not for physical training. Note that like the acquisition process, the consolidation process of temporal and spatial components of the task differed between physical and motor imagery practices. Indeed, we observed a decrease in movement duration between the Pretest and the Post24h for both groups, though this decrease was significantly greater for the PT compared to the MIT group, while the error rate slightly decreased after mental practice and slightly increased after physical practice.

The different levels of skill performance achieved after acquisition could not explain the different consolidation processes between the two practices. Indeed, the first control experiment revealed that for almost similar acquisition gains, consolidation still differed between the two training methods. In addition, differences in acquisition and consolidation processes between physical and motor imagery practice cannot be explained by indirect factors, such as sleep quality or fatigue, because these parameters did not differ between groups. Note also that before each test session all groups carried out two warm-up trials. Although we cannot ignore a possible warm-up effect at the beginning of the training phase for the physical group, we consider that learning largely overrides such an effect and definitely contribute to the different acquisition and consolidation processes between groups. Lastly, increasing the number of imagined trials (experiment 2) did not qualitatively change the consolidation process.

Our findings suggest that through consolidation a new initially fragile memory after motor imagery training is transformed into a stable memory. At the neurophysiological level, several studies have identified the cerebellum as the neural site of rapid learning and the primary motor cortex as the neural site of consolidation^70–72^. At the functional level, the availability of internal predictions only to drive the controller, may lead to a slow motor learning process that necessitates the passage of time to be consolidated. On the contrary, self-supervised learning during physical training, that is the use both internal predictions and sensory feedback to improve the controller, may lead to a rapid acquisition process with complete consolidation. In addition, it is possible that there was a saturation during physical training, meaning that potential benefits of consolidation have already been achieved through physical practice, leaving little opportunity to enhance skill further during consolidation.

Differences in motor learning processes between mental and physical practice has been reported in the literature in a long-term scale. For example, a previous study showed that five days of motor imagery practice could lead to the same amount of learning as after five-days of physical practice only if participants of the motor imagery practice group could physically practice for 2 h at the end of the motor imagery practice^73^. In another study, despite similar amount of training (5 days), motor imagery training did not drive robust changes on motor performance and brain activation as physical training^74^. How the passage of time consolidates skill learning after mental practice is an important question and needs further investigation. Is, for example, the passage of time the crucial parameter of such a consolidation, or is physical practice on the same task necessary to consolidate what has been learned during motor imagery training?

In the literature, motor imagery is often described as a cognitive process that engages neural structures, such as the parietal cortex and the prefrontal cortex^24,75^, encoding the goal of the movement^12,76^. Therefore, a positive effect of sleep on off-line improvement after motor imagery practice could be expected (see above, Robertson 2009; Cohen et al. 2005). Indeed, Debarnot and colleagues reported the positive influence of a nap^45^ or a night's sleep^43,44^ on motor performance improvement after a motor imagery training session. We propose that what it matters for consolidation after motor imagery practice is not the motor imagery process per se, but its content. When mental training implies larger goal-based components^43–45^, off-line gains could be observed after a period of sleep. On the other hand, motor imagery training in task with large motor skill components may consolidate through the passage of time.

Overall, the findings of the current study revealed different acquisition and consolidation processes after physical or motor imagery training. The key finding of this study is that after a consolidation period at least 6 h, motor imagery practice induces similar gains in motor skill than physical practice. In this way, imagery training may have high potential for motor rehabilitation.
